# Reconstruction of the Genomes of Drug-Resistant Pathogens for Outbreak Investigation through Metagenomic Sequencing

**DOI:** 10.1128/mSphere.00529-18

**Published:** 2019-01-16

**Authors:** Andre Mu, Jason C. Kwong, Nicole S. Isles, Anders Gonçalves da Silva, Mark B. Schultz, Susan A. Ballard, Courtney R. Lane, Glen P. Carter, Deborah A. Williamson, Torsten Seemann, Timothy P. Stinear, Benjamin P. Howden

**Affiliations:** a Microbiological Diagnostic Unit Public Health Laboratory, Department of Microbiology and Immunology at the Peter Doherty Institute for Infection and Immunity, University of Melbourne, Melbourne, Victoria, Australia; b Doherty Applied Microbial Genomics, Department of Microbiology and Immunology at the Peter Doherty Institute for Infection and Immunity, University of Melbourne, Melbourne, Victoria, Australia; c Department of Infectious Diseases, Austin Health, Heidelberg, Victoria, Australia; d Melbourne Bioinformatics, University of Melbourne, Melbourne, Victoria, Australia; Escola Paulista de Medicina, Universidade Federal de São Paulo

**Keywords:** *Klebsiella pneumoniae*, antimicrobial resistance, metagenomics, microbiome, vancomycin-resistant enterococci

## Abstract

Culture-independent methods that target genome fragments have shown promise in identifying certain pathogens, but the holy grail of comprehensive pathogen genome detection from microbiologically complex samples for subsequent forensic analyses remains a challenge. In the context of an investigation of a nosocomial outbreak, we used shotgun metagenomic sequencing of a human fecal sample and a neural network algorithm based on tetranucleotide frequency profiling to reconstruct microbial genomes and tested the same approach using rectal swabs from a second patient. The approach rapidly and readily detected the genome of Klebsiella pneumoniae carbapenemase (KPC)-producing K. pneumoniae in the patient fecal specimen and in the rectal swab sample, achieving a level of strain resolution that was sufficient for confident transmission inference during a highly clonal outbreak. The analysis also detected previously unrecognized colonization of the patient by vancomycin-resistant Enterococcus faecium, another multidrug-resistant bacterium.

**IMPORTANCE** The study results reported here perfectly demonstrate the power and promise of clinical metagenomics to recover genome sequences of important drug-resistant bacteria and to rapidly provide rich data that inform outbreak investigations and treatment decisions, independently of the need to culture the organisms.

## Abstract (precis)

The study results reported here perfectly demonstrate the power and promise of clinical metagenomics to recover genome sequences of important drug-resistant bacteria and to rapidly provide rich data that inform outbreak investigations and treatment decisions, independently of the need to culture the organisms.

## INTRODUCTION

Clinical and public health microbiology is undergoing a major transformation driven largely by high-throughput microbial whole-genome sequencing (WGS) (1). The application of microbial genomics in clinical bacteriology, including high-resolution microbial characterization, outbreak investigation for nosocomial and public health pathogens, and antimicrobial resistance (AMR) detection and prediction, has been well described (2–6). However, such applications generally relied on routine culture methods to isolate the pathogens of interest, selecting a single or few colonies from culture for sequencing. That approach presents a potential bias for subsequent analyses and does not allow characterization of pathogens that are unculturable, that occur in concentrations below the level of culture detection, or that are unsuspected in a clinical sample.

Culture-independent genomic approaches, such as metagenomic sequencing, are promising tools that overcome these difficulties by direct characterization of pathogens from clinical samples (7–9). Metagenomic approaches have been shown to complement culture-based techniques for epidemiological typing and for detecting antimicrobial resistance (AMR) genes (10–12). Current literature on clinical metagenomics is largely based on interrogating the metagenome at “first-order”-level analyses; that is, characterizing bacterial biodiversity at the 16S rRNA gene level, identifying the functional profile of the microbial community, and/or selectively identifying a single pathogen of interest (13–15). However, although rudimentary typing information can be gleaned (16), obtaining strain-level resolution data from metagenomic sequencing beyond the family level has remained a challenge (17), limiting clinical applications in outbreak investigation, public health surveillance, and accurate antimicrobial resistance profiling.

Recent metagenomic advances in environmental microbiology and ecology have shown an improved capacity to differentiate strains of the same species through advanced binning techniques such as the use of tetranucleotide frequency profiles projected onto emergent self-organizing maps and the use of expectation maximization algorithms to optimize gene reconstruction (18–24). These methods have facilitated the more complete and accurate reconstruction of individual genomes from metagenomic data sets derived from ecological samples (7, 22).

Here, in this proof-of-principle study, we investigated whether these modern approaches to metagenomic analysis and bacterial genome reconstruction could be applied for the clinical purposes of infection control and outbreak investigation through metagenomic sequencing of samples from patients with long-term colonization with an extensively drug-resistant pathogen—Klebsiella pneumoniae carbapenemase (KPC)-producing K. pneumoniae.

## RESULTS

**FIG 1:**
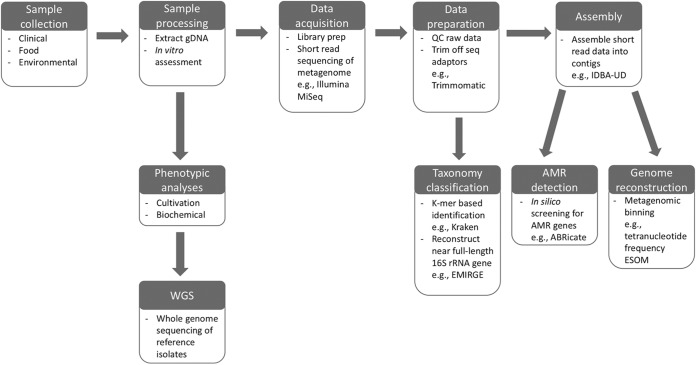
Schematic of the metagenomic workflow employed in this study. Further details can be found in Methods and Methods. Boxes labeled “Phenotypic analyses” and “WGS” represent routine diagnostics and microbial genomics, respectively.

The global approach to our metagenomic workflow for identifying antibiotic-resistant pathogens (and AMR genes) directly from primary samples is shown in Fig. 1. Briefly, whole-community genomic DNA (gDNA) was extracted from clinical samples, prepared for high-throughput sequencing, and processed for downstream bioinformatic analyses. Concurrent phenotypic assays were also conducted to support the metagenomic results.

### Sequencing yield.

Short-read sequencing of the metagenome of “patient A” produced 22,753,558 reads with an average Phred score of 33.9 (see Table S1 in the supplemental material). The number of assembled contigs greater than 1,000 bp in length was 12,051—from an initial total of 113,623 nonhuman contigs—with an average length of 5,896 bp and an *N*_50_ value of 13,307 (Table S2).

10.1128/mSphere.00529-18.4 TABLE S1 Sequence data for patient A, and patient B, metagenomic FASTQ files. Download Table S1, PDF file, 0.03 MB. Copyright © 2019 Mu et al. 2019 Mu et al. This content is distributed under the terms of the Creative Commons Attribution 4.0 International license.

10.1128/mSphere.00529-18.5 TABLE S2 Sequence data for patient A metagenomic assemblies using IDBA-UD. Download Table S2, PDF file, 0.03 MB. Copyright © 2019 Mu et al. 2019 Mu et al. This content is distributed under the terms of the Creative Commons Attribution 4.0 International license.

### *In silico* detection of AMR genes.

Table 1 highlights the AMR genes detected (representing the fecal resistome) with 100% base pair coverage in the metagenome, including genes for the following antibiotic classes: β-lactams, glycopeptides, macrolides, aminoglycosides, and tetracyclines. The *bla_KPC_* carbapenemase gene was detected at 100% coverage and nucleotide identity. The data also revealed the presence of the full *vanB* operon with 100% coverage, including *vanR-B*, *vanS-B*, *vanY-B*, *vanW-B*, *vanH-B*, *van-B*, and *vanX-B*, which collectively code for vancomycin resistance in Enterococcus faecium (vancomycin-resistant enterococci [VRE]). However, given that *bla_KPC_* can be carried on plasmids in several species of Enterobacteriaceae and that the *vanB* operon can be found in commensal anaerobic gut bacteria (25), we sought to confirm the presence of KPC-producing K. pneumoniae and *vanB* VRE through reconstruction of 16S rRNA genes and more-complete bacterial genomes from the metagenomic sequence data.

**TABLE 1:** Detection of antimicrobial resistance genes in patient A metagenome using ABRicate^*a*^

Contig^*b*^	Start (bp)	End (bp)	Gene^*c*^	Identity (%)	GenBank accession no.
FM_4827	2,150	3,076	*sul1_2*	100	CP002151
FM_1035	1,0808	11,689	*blaKPC-2_1*	100	AY034847
FM_1074	7,275	8,135	*blaSHV-12_1*	100	AF462395
FM_10044	186	1,001	*aph*(*3′*)*-Ia_1*	100	V00359
FM_452	2,2060	22,866	*vanY-B_1*	100	AF192329
FM_784	1,5763	16,260	*dfrG_1*	100	AB205645
FM_313	6,894	10,046	*oqxB_1*	100	EU370913
FM_313	1,0070	11,245	*oqxA_1*	100	EU370913
FM_2352	2,933	4,853	*tet*(*W*)*_4*	99.9	AJ427422
FM_10870	34	873	*blaOXA-9_2*	99.9	JF703130
FM_9051	161	1,381	*tet*(*40*)*_1*	99.8	FJ158002
FM_7453	1,068	1,565	*dfrA12_1*	99.8	AB571791
FM_12072	99	965	*aadE_1*	99.8	KF864551
FM_452	2,0546	21,889	*vanS-B_1*	99.6	AF192329
FM_452	2,3708	24,679	*vanH-B_1*	99.4	AF192329
FM_452	1,9884	20,546	*vanR-B_1*	99.2	AF192329
FM_452	2,4672	25,700	*vanA-B_1*	98.9	AF192329
FM_137	6,369	7,847	*msr*(*C*)*_1*	98.9	AY004350
FM_255	25,338	25,757	*fosA_3*	98.6	NZ_ACWO01000079
FM_452	22,884	23,711	*vanW-B_1*	97.6	AF192329
FM_452	25,706	26,314	*vanX-B_1*	96.7	AF192329

a Data represent 100% coverage with no gaps.

b In the designations in column 1, “FM” represents the fecal metagenome of patient A and the number that follows “FM” represents the associated metagenomic contig number.

c ResFinder database.

### Taxonomy of metagenomic reads.

Near full-length 16S rRNA genes were reconstructed for both K. pneumoniae and E. faecium from the gut microbial community of fecal samples from patient A. The EMIRGE (Expectation Maximization Iterative Reconstruction of Genes from the Environment) (24) program reconstructed 15 16S rRNA genes, in which a K. pneumoniae 16S rRNA gene was recovered with 100% nucleotide identity over 1,161 bp (Table 2). A near complete 16S rRNA gene (1,344 bp) was reconstructed and classified as E. faecium at 100% nucleotide identity. An independent *k*-mer-based approach (Kraken v0.10.5-beta) (26) also suggested the presence of K. pneumoniae (7.42% read assignment) and E. faecium (3.82% read assignment) from a total of 98.9% metagenomic reads (pre-removal of host-derived sequences) that were classified as bacterial in patient A’s fecal sample.

**TABLE 2:** 16S rRNA genes from patient A metagenome reconstructed using EMIRGE

Taxon	Length seq DB (bp)^*d*^	Length inferred seq (bp)^*e*^	Nucleotide ID^*f*^	Identity (%)
Akkermansia muciniphila	1,434	1,500	1431	99.8
Bacteroides cellulosilyticus	1,193	1,202	1183	99.2
Bacteroides uniformis	1,145	1,145	1143	99.8
Bacteroides uniformis	680	680	676	99.4
Clostridium bolteae	1,309	1,309	1305	99.7
Clostridium glycyrrhizinilyticum	1,268	1,266	1231	97.1
Dialister invisus	1,290	1,302	1287	99.8
Enterococcus faecium	1,344	1,344	1344	100
Eubacterium dolichum	1,484	1,516	1381	93.1
Klebsiella pneumoniae	1,161	1,161	1161	100
Lactobacillus pentosus	1,468	1,468	1467	99.9
Parabacteroides merdae	1,376	1,376	1376	100
Uncultured bacterium^*a*^	1,092	1,092	1092	100
Uncultured organism^*b*^	1,121	1,121	1121	100
Uncultured organism^*c*^	1,191	1,191	1191	100

a *Bacteroidetes*/*Bacteroides*.

b *Firmicutes*/*Clostridium* XIVa.

c *Proteobacteria*/*Sutterellaceae*.

d Length seq DB, length of 16S rRNA gene contained in the Ribosomal Database Project.

e Length inferred seq, length of 16S rRNA gene reconstructed using EMIRGE.

f Nucleotide ID, the number of nucleotides from the inferred seq matching the length seq DB.

### Reconstruction of bacterial genomes from metagenomic reads.

**FIG 2:**
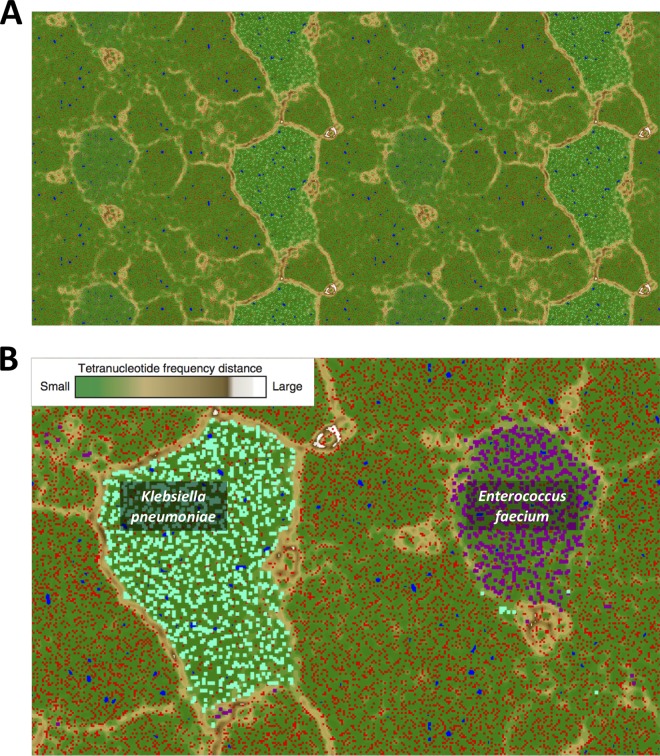
Emergent self-organizing maps (ESOMs) of the tetranucleotide frequencies of patient A contigs (A) in binned genomes representing Klebsiella pneumoniae and Enterococcus faecium (B). Tetranucleotide frequency profiles of DNA fragments between 2.5 kb to 5 kb in length are projected onto the ESOM. A green background indicates small tetranucleotide frequency distances and a white background large tetranucleotide frequency distances, while contigs derived from the patient A microbiome are highlighted in red, Klebsiella pneumoniae (GenBank accession no. CP008797) in teal, and a *vanB*-carrying E. faecium strain (Aus0085; GenBank accession no. NC_021994.1) in purple. The remaining metagenomic bins represent commensal organisms in patient A gut microbiome.

Emergent self-organizing maps (ESOMs) of the tetranucleotide frequencies of patient A-derived metagenomic contigs reconstructed discrete genome “bins” of organisms from the gut microbial community (Fig. 2A), with distinct K. pneumoniae and E. faecium bins observed (Fig. 2B). BLAST analysis of the assembled metagenome contigs associated with Klebsiella pneumoniae identified the presence of *bla*_KPC_, with mapping of the *bla*_KPC_-carrying contig (15,061 bp) to AUSMDU00008119 returning 100% pairwise sequence identity to the *bla*_KPC_ plasmid in AUSMDU00008119 (see Fig. S1 in the supplemental material). Furthermore, the metagenome-derived *bla_KPC_*-carrying contig encompasses the full transposon element, Tn*4401*.

10.1128/mSphere.00529-18.1 FIG S1 Comparison of Klebsiella pneumoniae transposon element Tn*4401* data. PacBio AUSMDU00008119 KPC plasmid 1 (outer ring) was used as the reference to provide a genomic context for (i) *bla_KPC2_* (red; GenBank accession no. AY034847.1); (ii) a reference K. pneumoniae transposon element, Tn*4401* (blue; BioSample accession no. SAMN07452764); and (iii) a patient A metagenome-derived, *bla_KPC2_* carrying contig (green). Download FIG S1, PDF file, 1.2 MB. Copyright © 2019 Mu et al. 2019 Mu et al. This content is distributed under the terms of the Creative Commons Attribution 4.0 International license.

### *In silico* molecular typing.

Analysis of the assembled metagenomic contigs from patient A identified the presence of complete multilocus sequence typing (MLST) profiles for the organisms of interest as ST258 K. pneumoniae and both ST555 and ST796 E. faecium. In comparison, *in silico* MLST based on whole-genome sequences of the cultured isolates from patient A fecal samples identified ST258 K. pneumoniae and an ST796 E. faecium sequence (AUSMDU00010620). Comparisons between metagenomic and isolate genome sequences for E. faecium revealed a single base substitution in a *pstS* allele of the E. faecium MLST scheme as the reason for the differences in sequence types (Fig. S2; see also Table S3). Further analysis of the metagenomic reads using read mapping highlighted the presence of the two *pstS* alleles at bp 96 in equal proportions, supporting the idea of the colonization of patient A with both VRE sequence types.

10.1128/mSphere.00529-18.2 FIG S2 Comparison of vancomycin-resistant Enterococcus faecium genomes. E. faecium AUS0085 (GenBank accession no. NC_021994.1) was used as the reference to compare a VRE isolate cultured from a fecal sample (dark red; AUSMDU00010620) from primary patient A and patient A metagenome-derived, E. faecium-associated contigs (green). Download FIG S2, PDF file, 1.2 MB. Copyright © 2019 Mu et al. 2019 Mu et al. This content is distributed under the terms of the Creative Commons Attribution 4.0 International license.

10.1128/mSphere.00529-18.6 TABLE S3 *In silico* multilocus sequence typing of isolate genomes and organism metagenomes. Download Table S3, PDF file, 0.07 MB. Copyright © 2019 Mu et al. 2019 Mu et al. This content is distributed under the terms of the Creative Commons Attribution 4.0 International license.

### Inference of transmission.

**FIG 3:**
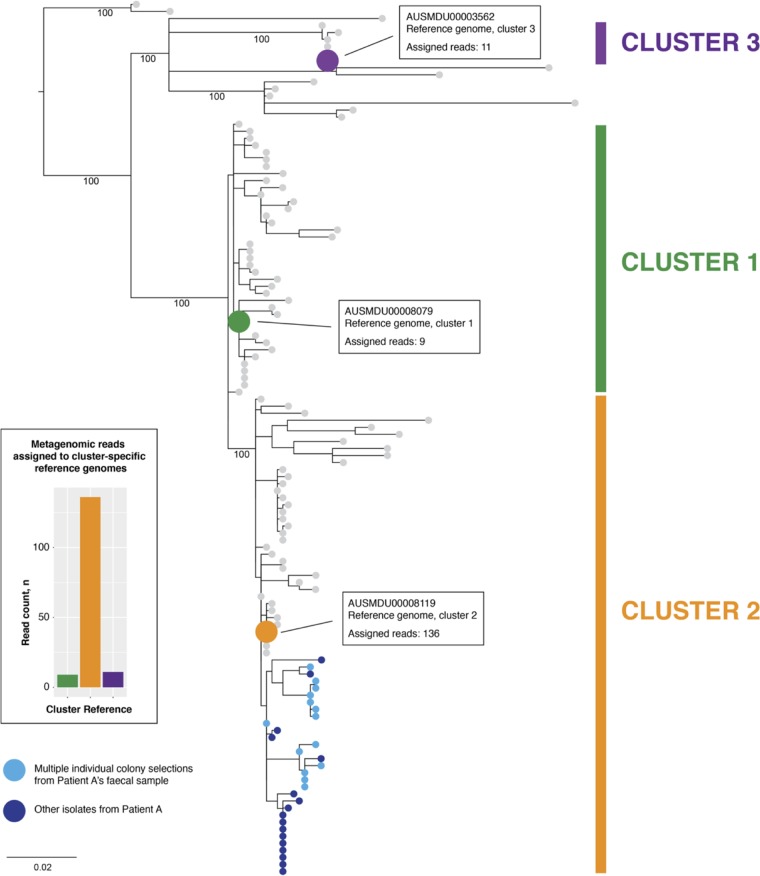
Transmission inference from metagenomic reads. A maximum likelihood phylogenetic tree of a local multi-institutional outbreak of KPC-producing K. pneumoniae is shown, with three transmission clusters defined through combined genomic and epidemiological analyses (38). Bootstrap values from 1,000 replicates trees are shown for major phylogenetic branches. Reference genomes of representative isolates are colored according to transmission cluster as follows: purple, AUSMDU00003562; green, AUSMDU00008079; orange, AUSMDU00008119. Individual colony sequencing data from 32 isolates (including 14 from the fecal sample used in this study) obtained from six clinical samples from patient A over 8 months are represented in blue and are shown to have been phylogenetically derived from cluster 2. The histogram on the left shows the number of metagenomic reads assigned to each representative reference genome, indicating the closest match to the cluster 2 reference genome (AUSMDU00008119).

Using a custom Kraken database, we determined that the genes in patient A’s colonizing ST258 K. pneumoniae population were most closely related to the reference genome from transmission cluster 2 (AUSMDU00008119), suggesting that the patient A-derived K. pneumoniae isolate was most likely linked to this transmission network (Fig. 3). Among the local reference genomes, AUSMDU00008119 had 136 metagenomic reads assigned compared to 11 and 9 reads for the other local cluster reference K. pneumoniae isolate genomes.

**FIG 4:**
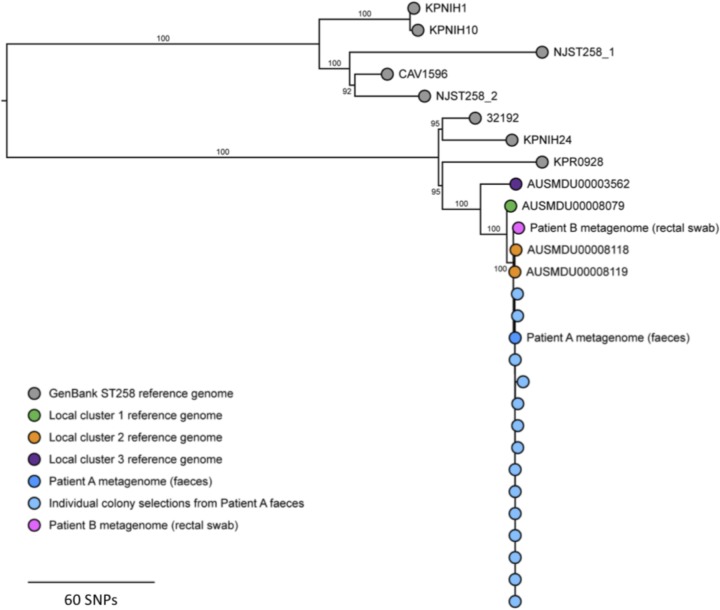
Phylogenetic comparison of metagenomic reads to reference genomes. Data represent results of phylogenetic comparisons of metagenomic data from sequencing of patient A’s fecal sample and patient B’s rectal swab, together with local representative reference genomes from a statewide, multi-institutional outbreak of ST258 KPC-producing K. pneumoniae and eight publicly available ST258 reference genomes available in GenBank. Genomes from sequencing of individually selected K. pneumoniae colonies derived from patient A’s fecal sample are also shown. A maximum likelihood tree was inferred from core-genome single nucleotide polymorphism sites and was midpoint rooted. Branch labels indicate support from 1,000 bootstrap replicates. The scale bar represents an approximate value.

This transmission inference was corroborated by phylogenetic comparisons with GenBank and local reference genomes using the core genome single nucleotide polymorphism (SNP) sites in the metagenomic data(Fig. 4). These analyses suggested that patient A’s sample harbored isolates that closely resembled the reference genomes from cluster 2 compared to the local and international ST258 reference genomes. Additionally, sequences of the individual K. pneumoniae colonies derived from patient A’s fecal sample most closely matched the metagenomic sequences from patient A. These individual colony genomes differed from each other by no more than 27 SNPs (Fig. 4.)

These inferences from the metagenomic analyses were also supported by epidemiological hypotheses. The majority of patients colonized with KPC-producing K. pneumoniae comprising cluster 2 had been hospitalized at the same tertiary hospital as patient A, thus providing a plausible epidemiological link to the transmission network at that hospital, negating the need to intensively investigate contacts of patient A who had been recently hospitalized elsewhere. Limited screening of these contacts did not find any further colonization with KPC. These data demonstrate the potential of clinical metagenomics to guide source tracking and infection control efforts.

### Reproducibility from rectal swabs.

The findings from patient A led to efforts to derive similar data from metagenomic sequencing of rectal swabs. A total of 223,247,382 reads with an average Phred score of 32.7 and 161,017,172 reads with an average Phred score of 32.2 were derived from patient B’s culture-“negative” (AUSMDU00016469) and culture-“positive” (AUSMDU00016470) rectal swabs, respectively (Table S1). Assembled contigs were filtered to remove those <1,000 bp, in which 45,596 contigs remained with an average length of 4,200 bp and an *N*_50_ of 8,804 for sample AUSMDU00016469 and 40,442 contigs with an average length of 4,870 and an *N*_50_ of 13,726 for sample AUSMDU00016470.

*bla*_KPC_ was detected at 100% coverage and nucleotide identity from AUSMDU00016470. Additionally, 39,280 reads were mapped to the Tn*4401* transposon carrying *bla*_KPC_, while a ST258 K. pneumoniae genome was reconstructed from AUSMDU00016470. In contrast, only six reads from the carbapenemase-negative sample AUSMDU00016469 aligned (poorly) to Tn*4401*, with numerous mismatches. Together with the inability to identify a ST258 K. pneumoniae in this sample, this supported the idea of the absence of a KPC-producing K. pneumoniae strain in AUSMDU00016469.

As with the transmission inferences for patient A’s sample, the metagenomic data derived from patient B’s positive rectal swab (AUSMDU00016470) suggested that patient B harbored KPC-producing isolates that also were derived from the local transmission cluster 2 (Fig. 4).

## DISCUSSION

In this proof-of-principle study, we used modern metagenomic approaches to derive detailed information from analyses of fecal and rectal swab samples of patients colonized with multidrug-resistant health care-associated organisms—representing crucial threats to hospital infection control. Furthermore, we demonstrated not only that these data could be used to inform outbreak investigations but also that metagenomic approaches could provide additional and potentially more accurate data for these investigations by capturing the within-host diversity of a pathogen under surveillance that is lost with single-isolate testing and sequencing. Although significant advances have been made in taxonomic assignment from metagenomic sequencing data, high-level discrimination beyond the family level has proved challenging, irrespective of the approaches used (17). Here, we employed tetranucleotide frequency profiling as the main strategy to reconstruct genomes of community members directly from a fecal specimen and to determine the presence of KPC as well as to identify previously unrecognized colonization with *vanB* VRE.

Tetranucleotide profiles (frequencies of the 256 combinations of 4-mers in each contig) represent a fundamental characteristic of DNA (27–29). Contigs with similar tetranucleotide profiles are derived from the same isolate, and therefore, projection of the frequency profiles of metagenomic contigs onto ESOMs can reconstruct genomes independently of reference-based alignment approaches, such as BLAST and/or read alignment. This highlights the potential to allow strain-level molecular characterization even when a reference isolate is not available *a priori* (7, 30). Encouragingly, we found that the number of reconstructed genomes (i.e., “bins”) roughly correlated with the number of 16S rRNA genes recovered in our EMIRGE analysis. The fact that this was achievable using a fecal sample and rectal swabs further speaks to the validity of this approach, given the microbial complexity of this sample type. The implication is that we can expect even greater levels of detail in analyzing less-complex and yet more clinically important samples such as blood or cerebrospinal fluid, provided adequate microbial sequence data can be obtained.

The clinical implications of the use of metagenomic approaches in outbreak investigations are 3-fold. First, such approaches allow detection of multiple pathogens that may not have been initially considered for testing or that require separate tests for identification. In this study, we performed metagenomics analyses to identify KPC-producing K. pneumoniae in the context of a local outbreak and unexpectedly detected cocolonization with *vanB* VRE. This has important implications for infection control issues that might otherwise have been missed—for example, due to co-colonization of multiple multidrug-resistant organisms, this patient would not be cohorted with other patients colonized with the local outbreak KPC strain. Second, the use of metagenomics in such approaches minimizes some of the bias inherent in selecting a single colony or a few colonies for WGS. In detecting *vanB* VRE, we were able to identify multiple populations of VRE with different sequence types by MLST. In contrast, our efforts to confirm the presence of VRE by culture on selective media identified only the ST796 VRE isolate, due to selection of a single colony. Here, had we been conducting an outbreak investigation for VRE, the importance of detecting multiple within-host populations would be clear. Finally, metagenomic approaches represent a promising method to undertake molecular comparisons of isolates for outbreak investigation independently of the need for culture, especially in instances where the target microorganism might not be readily independently cultured *in vitro*. Although this might not be frequently required, it might be important for pathogens where molecular or serological detection is currently or becoming routine practice, due to the difficulty in performing culture (e.g., Treponema pallidum) or to the convenience of modern molecular diagnostics (e.g., in the case of diarrheal pathogens such as Salmonella enterica or of sexually transmitted organisms such as Neisseria gonorrhoeae). It might also be important for outbreak investigation and surveillance in cases where antibiotics might have affected the yield from culture methods, necessitating diagnosis through molecular tests, as sometimes occurs with invasive meningococcal disease or listeriosis. In this study, we were able to obtain data from metagenomic sequencing of rectal swabs—which are often more conveniently obtained and widely used as screening samples in hospital infection control—that were comparable to the data obtained from sequencing of a fecal sample.

In considering these applications, there are several caveats with respect to the use of metagenomics in clinical settings. The optimal quality control metrics for metagenomic sequencing—in particular, the minimum required depth and coverage of sequencing across the pathogen genome of interest—have not been established. Some public health authorities have stipulated minimum and preferred sequencing depth requirements for WGS (31). However, with metagenomic sequencing, these depths are rarely achieveable without great expense. Host DNA depletion has been used to enrich for pathogen DNA in clinical samples in both clinical metagenomics and microbiome studies to improve the results, though host DNA depletion can result in some loss of pathogen DNA (32) The ability to identify mixed populations becomes increasingly challenging with reductions of sequencing depth, due to the inherent error rates encountered with next-generation sequencing. In our metagenomic data, we identified two VRE sequence types that differed by a single base substitution, though the reads conferring evidence arguing for and against the substitution were present in equal proportions. While ST555 and ST796 differ by only a single substitution in their MLST profile, they comprise distinct monophyletic clades at the core genome level and are common sequence types currently circulating in Australian health care facilities (33). The infection control practice of cohorting patients colonized with *vanB* VRE in local hospitals adds plausibility to evidence of the presence of both sequence types in our metagenomic data.

Although we assessed only one fecal specimen in this study, the richness of microbial characterization obtained using an alignment-free approach uncovered unrecognized colonization with vancomycin-resistant Enterococcus faecium (VRE), another high-risk antimicrobial-resistant pathogen. Furthermore, the availability of local, completely assembled reference genomes enhanced our ability to resolve transmission of a local KPC-producing K. pneumoniae outbreak, and while such analyses can be expensive to perform, we would argue that the availability of high-quality local reference genomes would enhance any local outbreak genomics investigation. Genome-resolved metagenomics currently requires manual curation, but the analysis steps of clinical importance are amenable to automation, including tetranucleotide frequency profiling, thus adding to the current literature establishing the use of metagenomic sequencing as a viable technique in the context of clinical microbiology (34–37). With improving costs, sequencing yield, and bioinformatic approaches to analysis, metagenomics is poised to play a key role in the future of clinical and public health microbiology.

Our report presents as a proof-of-principle-like framework for using whole-community metagenomics as a culture-independent method to profile a patient colonized with multidrug-resistant pathogens, here achieving sufficient strain-level resolution to assist with an outbreak investigation. This study adds to the increasing metagenomic applications in clinical and public health microbiology.

## MATERIALS AND METHODS

### Epidemiological context.

One fecal sample collected from a patient (patient A) with known KPC-producing K. pneumoniae colonization underwent whole-community metagenomics (Fig. 1). This particular sample was selected for shotgun metagenomics for three key reasons: (i) we were able to obtain multiple individual KPC-producing K. pneumoniae isolates from the sample for comparison, (ii) we had comprehensive metadata (including epidemiological data) associated with this patient (named “patient A” from a previous study (38), and (iii) fecal material represents a snapshot of the diverse and complex gut microbiome. In addition, patient A had not reported any recent travel but had been admitted to a tertiary hospital 2 months prior to sample collection. Following hospital admission, patient A had also been in close contact with several patients who had been recently admitted to several hospitals in the region.

### Ethics statement.

Data and samples were collected as part of an outbreak investigation through the Victorian Department of Health and Human Services under the Public Health and Wellbeing Act 2008 (https://www2.health.vic.gov.au/about/legislation/public-health-and-wellbeing-act). Ethics approval for metagenomics sequencing of human clinical samples was obtained through the Austin Health Human Research Ethics Committee (approval no. HREC/15/Austin/396), and informed consent was obtained from patients as part of this protocol.

### Whole-community genomic DNA extraction and high-throughput metagenomic sequencing.

Whole-community genomic DNA (gDNA) was extracted from 0.2 g of patient A’s feces using a QIAamp DNA stool kit following the manufacturer’s protocol with a mechanical lysis preprocessing step. Lysing Matrix B 2-ml tubes (MP Biomedicals) containing 0.1-mm-diameter silica beads were used for two 40-s cycles of mechanical lysis (Precellys 24 homogenizer; Bertin Technologies) at 6,000 rpm, with a 60-s rest on ice between the cycles. Dually indexed libraries were prepared from fecal genomic DNA and, together with a no-template control comprising nuclease-free water, were processed for sequencing using a Nextera XT kit (Illumina) and sequenced on a MiSeq system (V3; Illumina Inc., San Diego, CA, USA) (600 cycles) with a 1% (vol/vol) spike-in ratio of PhiX, following the manufacturer’s protocol.

### Culture-dependent whole-genome sequencing.

Concurrently, individual carbapenem-resistant K. pneumoniae colonies (*n* = 14) were picked at random from patient A’s primary sample plated on Brilliance CRE selective media (Thermo Fisher Scientific, Waltham, MA, USA). Genomic DNA from each colony was extracted using a JANUS automated workstation with a Chemagic viral DNA/RNA kit (PerkinElmer). Libraries were prepared as described above and sequenced on a NextSeq 500 system with 150-cycle paired-end chemistry as described in the manufacturer’s protocols (Illumina Inc., San Diego, CA, USA).

Representative KPC-producing K. pneumoniae isolates from previously defined local transmission networks were used as comparative genomes for this study (38). Three isolates were selected, all from different local transmission networks involving patients colonized or infected with KPC-producing K. pneumoniae, with the networks defined through combined genomic and epidemiological inference. DNA extraction, size selection, and sequencing on a Pacific Biosciences RS II system (Pacific Biosciences, Menlo Park, CA, USA) were performed as previously described (39), with additional Illumina sequencing data used for polishing to produce high-quality closed genomes.

Sequencing data from this study are available through the European Nucleotide Archive (BioProject PRJEB23689; see reference 38 for K. pneumoniae genomes).

### Bioinformatic analyses. (i) Reference genome assembly.

Reference genomes were assembled using Canu v1.5 (40), trimmed for overlapping ends (https://github.com/tseemann/berokka), and circularized. Illumina short-read data from the same gDNA sample were used to correct and polish the draft PacBio genomes using Pilon v1.22 (41) and Snippy v3.2 (https://github.com/tseemann/snippy). Further assembly of unmapped short-read data (i.e., Illumina reads that did not match chromosomal or larger plasmid DNA from PacBio-derived data) was performed using SPAdes v3.10.1 (42) to detect the presence of smaller plasmids potentially missed through DNA size selection. Prokka v1.11 was used to predict genes and assign predicted functions in the assembled genomes (43).

### (ii) Metagenomic sequence data processing.

Metagenomic data from patient A were processed prior to analysis with Trimmomatic v0.33 (44) for quality control and to remove adaptor sequences and PhiX contamination and to filter low-quality segments. Paired-end reads were interleaved and were assembled using Iterative de Bruijn Graph De Novo Assembler for Uneven sequencing Depth (IDBA-UD) (45) compiled for long (i.e., 651-bp) reads. Further quality control included removing host-derived sequences using DeconSeq and the Human Genome Reference Sequence (build 38; GCA_000001405.22) prior to downstream analyses (46).

### *In silico* detection of antimicrobial resistance genes and molecular typing.

Assembled metagenomic contigs and reference genome assemblies were screened for acquired antimicrobial resistance genes, including KPC genes, using a custom BLAST-based tool, ABRicate (https://github.com/tseemann/abricate), to search against the NCBI Bacterial Antimicrobial Resistance Reference Gene Database (https://www.ncbi.nlm.nih.gov/bioproject/PRJNA313047). Similarly, alleles were detected using a multilocus sequence typing (MLST) scheme and *mlst* (https://github.com/tseemann/mlst), representing another in-house BLAST tool to search against the entire reference database of MLST profiles (downloaded from https://pubmlst.org).

### Reconstructing 16S rRNA gene sequences.

Small-subunit 16S rRNA gene sequences were reconstructed from patient A unassembled short-read metagenomic data (after removal of host-derived sequences; AUSMDU00010683) using the Expectation Maximization Iterative Reconstruction of Genes from the Environment (EMIRGE v0.61.1) program (24). The following database and parameters were incorporated to compute over 80 iterations: the SILVA Small Subunit database (release 111 (47) (employed as a training reference set); length of reads, 151; insertion size, 683; standard deviation, 68; Phred score, 33. Reconstructed 16S rRNA genes were queried against the Ribosomal Database Project data (48) using BLAST. A *k*-mer-based approach, using Kraken v1.0 (26), classified unassembled read data to support EMIRGE results.

### Metagenomic binning.

To reconstruct bacterial genomes from the gut microbial community, an emergent self-organizing map (ESOM) was used. Tetranucleotide frequencies were calculated for the assembled contigs using Perl scripts developed by Dick et al. (20) in preparation for analysis using ESOMs. The primary map structure was determined using *in silico*-fragmented (>5-kb) contigs, while contigs between 2.5 kb and 5 kb in length were projected onto the ESOM using their tetranucleotide frequency profiles. Contigs with a native length below 2.5 kb were removed from analyses. Genomic binning was analyzed using the Databionic ESOM Tool (49) with default settings except as follows: K-Batch training algorithm in 200 × 400 windows, a starting value of 50 for the radius, and normalization of data points by RobustZT transformation. Cluster valleys were interpreted to indicate areas of high density (i.e., small tetranucleotide frequency distances), representing contigs derived from a single organism; while cluster boundaries of no/few data points (i.e., large tetranucleotide frequency distances) defined metagenomic “bins.”

Reference KPC genomes (AUSMDU00003562, AUSMDU00008079, and AUSMDU00008119; GenBank and BioSample accession numbers SAMN07503096, SAMN07452764, and SAMN07503088) and VRE genomes (Aus0085—GenBank accession no. NC_021994.1) were included in the ESOM analysis as positive controls for binning and were also used to guide taxonomic identification of “binned” genomes. KPC- and VRE-associated contigs derived from the metagenome were visually compared to their respective reference isolate genomes using CGView Comparison Tool (see Fig. S2 and S3 in the supplemental material) (50). These metagenome-derived contigs were also compared to draft genome assemblies of the isolates derived from the sample cultured on selective media. Sequence comparisons were computed by BLAST (51) and are visually presented in graphical maps (Fig. S2 and S3).

10.1128/mSphere.00529-18.3 FIG S3 Comparison of Klebsiella pneumoniae carbapenemase-producing K. pneumoniae genomes. PacBio AUSMDU00008119 was used as the reference genome to compare against patient A metagenome-derived, K. pneumoniae-associated contigs (green). Download FIG S3, PDF file, 2.1 MB. Copyright © 2019 Mu et al. 2019 Mu et al. This content is distributed under the terms of the Creative Commons Attribution 4.0 International license.

### Transmission cluster inference of KPC K. pneumoniae.

To determine the most likely transmission cluster source of K. pneumoniae for patient A, PacBio reference genomes (AUSMDU00003562, AUSMDU00008079, and AUSMDU00008119) from three previously defined local transmission clusters (38) were assembled using the methods described above and used to build a custom Kraken database. Whole-community metagenomic sequencing reads were analyzed in Kraken v0.10.5-beta using the custom database to identify the most closely related reference genome.

In parallel, the canonical core genome SNP sites used to infer the phylogeny of eight closed ST258 K. pneumoniae reference genomes from GenBank (accession numbers NZ_CP010361.1, NZ_CP011647.1, NZ_CP007727.1, NZ_CP008827.1, NZ_CP008797.1, NZ_CP008831.1, NZ_CP006923.1, and NZ_CP006918.1) and the local representative ST258 reference genomes were identified using Snippy v4.1.0 by aligning each genome to AUSMDU00008119. Illumina reads from sequencing of 14 individual colony selections from patient A’s fecal sample were also included. Next, metagenomic reads from patient A’s fecal sample were also aligned to AUSMDU00008119 using bwa mem (52). The predominant base call in the metagenome alignment at each of the identified core genome SNP sites was determined and extracted to append to the core genome SNP alignment using a custom script (https://github.com/kwongj/extract_core_snps). The resulting modified core SNP alignment comprising the GenBank and local reference genomes, the individual colony selection sequences, and the metagenomic database calls was used to infer a maximum likelihood phylogenetic tree using IQ-TREE (53).

### Metagenomic sequencing from rectal swabs.

Rectal swabs from a second patient (patient B) also underwent whole-community metagenomics. These included a “negative” swab—no growth on Oxoid Brilliance CRE selective agar (Thermo Fisher Scientific, Waltham, MA, USA)—prior to KPC-producing K. pneumoniae colonization, followed by a “KPC-positive” swab a month later. Patient B also reported no recent travel, and although patient B had no direct contact with patient A, patient B was epidemiologically linked to the same transmission network. Rectal FLOQSwabs (Copan Diagnostics) with visible fecal material from patient B were collected into ESwab Liquid Amies media. Following mechanical lysis, gDNA was extracted by the use of a QIAamp DNA stool kit and sequenced on a NextSeq system (Illumina Inc., San Diego, CA, USA) (300 cycles) using Nextera libraries. Metagenomic sequencing data processing and analysis were performed as described for the sample from patient A.

### Data availability.

Metagenomic sequence data from this study are available through the European Nucleotide Archive (BioProject PRJEB23689).
