# Supplementary material for: Reconstruction of the Genomes of Drug-Resistant Pathogens for Outbreak Investigation through Metagenomic Sequencing
Source: mSphere. 2019 Jan 16;4(1):e00529-18. doi: 10.1128/mSphere.00529-18 (PMC6336080; doi:10.1128/mSphere.00529-18)
Supplement: TABLE S1 [file mSphere.00529-18-st001.pdf]

Table S1. Sequence data for Patient A, and Patient B, metagenomic FASTQ files

Sample	Reads	Yield	GC%	Minimum length	Average length	Maximum length	Mode length	Phred	Average Quality
Patient A	22,753,558	6,355,932,790	45.1	35	279	301	301	33	33.9
Patient B_1	223,247,382	32,600,252,227	44.5	35	146	151	151	33	32.7
Patient B_2	161,017,172	23,806,327,128	51.2	35	147	151	151	33	32.2
