# Supplementary material for: Reconstruction of the Genomes of Drug-Resistant Pathogens for Outbreak Investigation through Metagenomic Sequencing
Source: mSphere. 2019 Jan 16;4(1):e00529-18. doi: 10.1128/mSphere.00529-18 (PMC6336080; doi:10.1128/mSphere.00529-18)
Supplement: TABLE S2 [file mSphere.00529-18-st002.pdf]

Table S2. Sequence data for Patient A metagenomic assemblies using IDBA-UD

Sample	No. contigs	Base pairs	Ok	Ns	Gaps	Minimum length	Average length	Maximum length	N50
Patient A	113,623	106,938,398	106,938,398	0	0	200	941	203,719	4,750
Patient A [#]	12,051	71,059,482	71,059,482	0	0	1,001	5,896	203,710	13,307

[#] Assembly file processed to remove contigs smaller than 1,000 bp
