# Supplementary figures and images for: Reconstruction of the Genomes of Drug-Resistant Pathogens for Outbreak Investigation through Metagenomic Sequencing
Source: mSphere. 2019 Jan 16;4(1):e00529-18. doi: 10.1128/mSphere.00529-18 (PMC6336080; doi:10.1128/mSphere.00529-18)

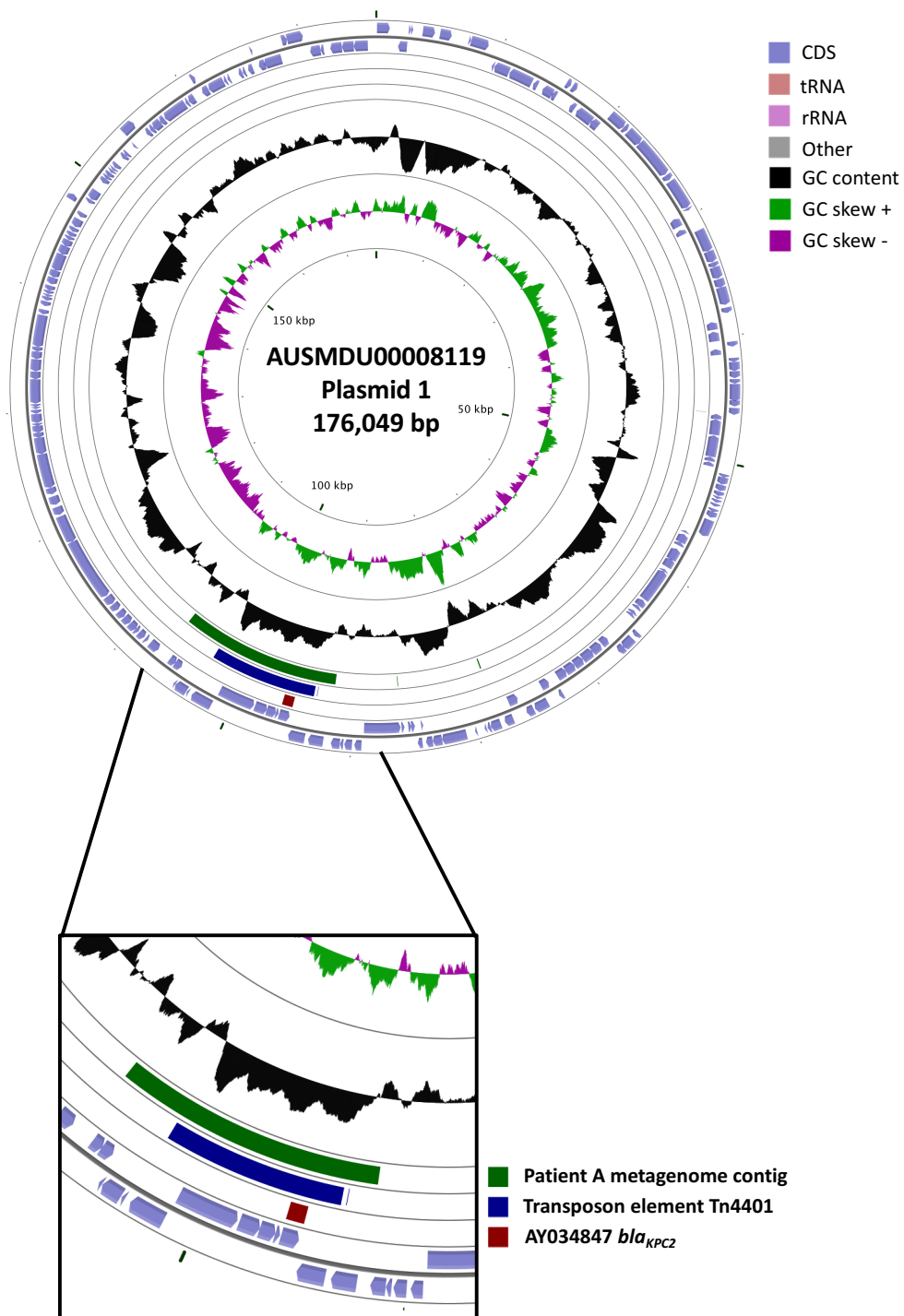

Supplement: FIG S1 [file mSphere.00529-18-sf001.pdf]

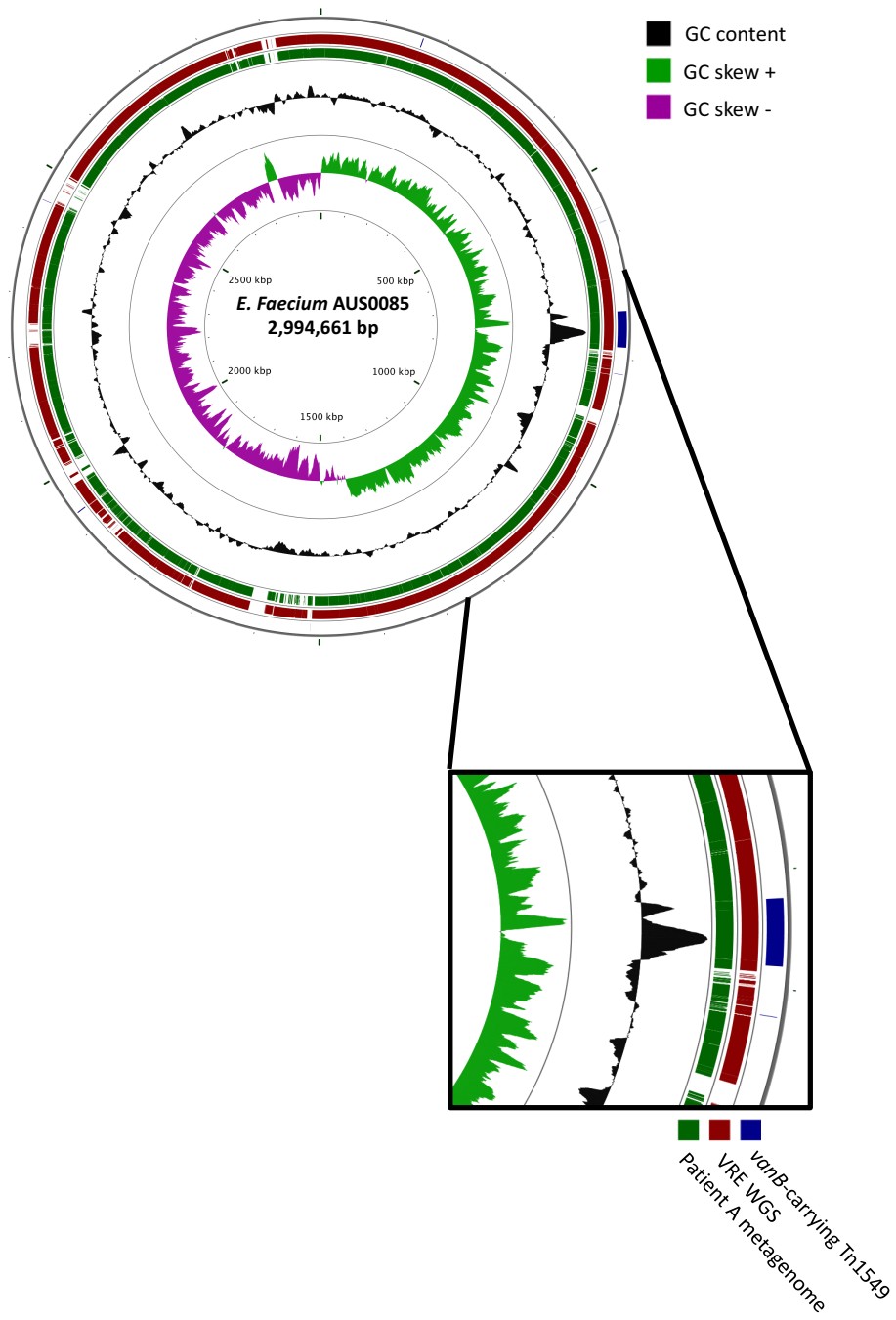

Supplement: FIG S2 [file mSphere.00529-18-sf002.pdf]

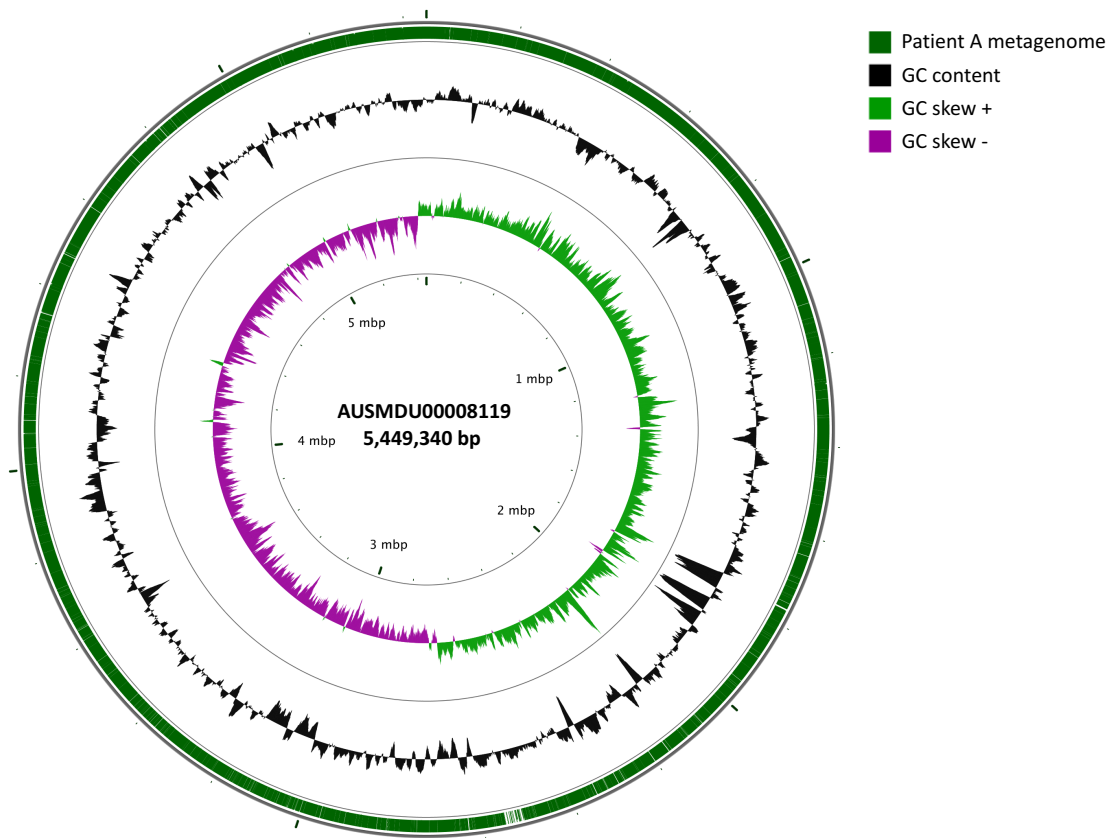

Supplement: FIG S3 [file mSphere.00529-18-sf003.pdf]
