# Supplementary material for: Reconstruction of the Genomes of Drug-Resistant Pathogens for Outbreak Investigation through Metagenomic Sequencing
Source: mSphere. 2019 Jan 16;4(1):e00529-18. doi: 10.1128/mSphere.00529-18 (PMC6336080; doi:10.1128/mSphere.00529-18)
Supplement: TABLE S3 [file mSphere.00529-18-st003.pdf]

Table S3 ***In silico*** **multi-locus sequence typing of isolate genomes and organism metagenomes**

**Sample**	**Scheme**	**ST**	***gapA***	***infB***	***mdh***	***pgi***	***phoE***	***rpoB***	***tonB***
AUSMDU00008119	*kpneumoniae*	258	3	3	1	1	1	1	79
KP metaG	*kpneumoniae*	258	3	3	1	1	1	1	79
			atpA	***ddl***	***gdh***	***purK***	***gyd***	***pstS***	***adk***
VRE*fm* WGS	*efaecium*	796	4	1	1	1	1	20	1
VRE*fm* metaG	*efaecium*	555	4	1	1	1	1	1	1
